# METTL3-mediated activation of Sonic Hedgehog signaling promotes breast cancer progression

**DOI:** 10.3389/fcell.2025.1674339

**Published:** 2025-10-01

**Authors:** Sunanda Baidya, Utpal Barua, Muntasim Rahman Shanto, Tayeba Sultana Sonia, Md. Al Amin, Saima Sultana, Nusrat Jerin, Khadiza Jahan, Israt Jahan, Shakera Ahmed, Mohammed Moinul Islam, Ramendu Parial, Muhammad Mosaraf Hossain, Abu Sadat Mohammad Noman

**Affiliations:** ^1^ Laboratory of Immune Signaling, Department of Biochemistry and Molecular Biology, University of Chittagong, Chattogram, Bangladesh; ^2^ EuGEF Research Foundation, Chattogram, Bangladesh; ^3^ Department of Biochemistry and Molecular Biology, University of Chittagong, Chattogram, Bangladesh; ^4^ Department of Surgery, Chittagong Medical College, Chattogram, Bangladesh; ^5^ Laboratory of RNA Biology and Molecular Oncology, Department of Biochemistry and Molecular Biology, University of Chittagong, Chattogram, Bangladesh

**Keywords:** breast cancer, sonic hedgehog developmental pathway, epigenetic regulation, methyltransferase like-3 (METTL3), N6-methyladenosine (m6A)

## Abstract

**Introduction:**

Breast cancer represents a heterogeneous group of tumors characterized by diverse molecular and clinical features, driven by both genetic alterations and epigenetic regulation. Among these mechanisms, the Hedgehog (Hh) developmental pathway, particularly elevated levels of its ligand Sonic Hedgehog (SHH), has been implicated in breast cancer progression. Methyltransferase-like 3 (METTL3), the core catalytic component of the m6A methyltransferase complex, responsible for N6-methyladenosine (m6A) modification of mRNA, has shown a stronger prognostic relevance in regulating mRNA stability and cancer development than other m6A writers, erasers, or readers. Despite evidence suggesting that both SHH and METTL3 contribute to tumor growth in breast tissue, the functional relationship between these factors remains unclear. In this study, we investigated the potential of the METTL3-SHH axis in breast cancer progression to address this gap.

**Methods:**

We have performed bioinformatic analyses by utilizing data from UALCAN, cBioPortal, and GEPIA platforms to comprehensively investigate the methylation patterns, gene expression levels, and mutation profiles of specific genes of interest. Expressions of METTL3 and components of the SHH signaling pathway were analyzed by qRT-PCR. Statistical analyses were performed by using Student’s t-test, Spearman and Pearson coefficient (r) test, ANOVA test, and log–rank test.

**Results:**

Analysis of 35 breast cancer patients of Bangladesh and gene expression data from The Cancer Genome Atlas (n = 1,021) database revealed METTL3 is overexpressed in breast cancer, and upregulation of METTL3 and downstream key components of the SHH signaling pathway (p < 0.05 vs. control) correlates significantly with worse patient outcomes (HR = 1.3). These findings suggest a possible regulatory mechanism linking METTL3-mediated m6A modification to SHH signaling in breast cancer progression. Elucidating this axis could provide novel insights into tumor biology and identify promising targets for epigenetic therapies.

## Graphical Abstract

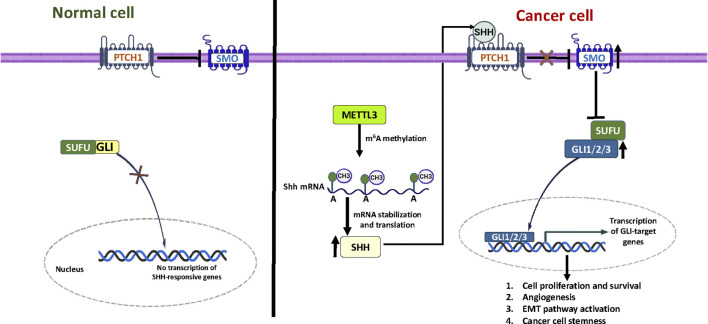

## 1 Introduction

Breast cancer (BC) is the most common malignancy among females worldwide, primarily arising in glandular tissue and accounting for approximately 15% of cancer-related deaths globally (Davari et al., 2017; Khan et al., 2017). Despite advancements in diagnosis and treatment, the incidence and mortality rates for breast cancer are rising in countries like Bangladesh. According to GLOBOCAN 2022, the age-standardized incidence rate for breast cancer in Bangladeshi women is approximately 15.2 per 100,000, and it remains the leading cancer in females in terms of both incidence and mortality (Bray et al., 2024). The classification of breast cancer into four phenotypic categories, Luminal A, Luminal B, HER2+, and TNBC, is critical for diagnosis and the development of subgroup-specific therapies aimed at improving clinical outcomes (Staaf et al., 2010). While established treatment options exist for estrogen receptor-positive (ER+), progesterone receptor-positive (PR+), and human epidermal growth factor receptor-positive 2 (HER2+) breast cancers, triple negative breast cancer (TNBC) remains particularly challenging due to its aggressive nature and the lack of effective targeted therapies (Marotti et al., 2017). The Hedgehog (Hh) signaling pathway has a significant role in influencing breast cancer progression. Among the three ligands, Sonic Hedgehog (SHH) is crucial for the ligand-dependent activation of this pathway (Noman et al., 2017). SHH can activate the pathway through two mechanisms: canonical and non-canonical signaling. In the canonical pathway, SHH interacts with and inhibits the transmembrane protein Patched (PTCH1), which normally suppresses the activity of Smoothened (SMO) (Choudhry et al., 2014; Rimkus et al., 2016). Once SMO is activated, it initiates a signaling cascade that causes the glioma-associated oncogene (GLI) family proteins to translocate into the nucleus. This process leads to the transcription of target genes such as Cyclin D1, Cyclin E, c-Myc, BCL-2, and SNAIL (Denef et al., 2000; Dahmane and Altaba, 1999). The transcription factor NF-κB regulates SHH expression by binding to a site within the SHH promoter’s CpG island. Studies have shown that decreased methylation of this region increases SHH expression in various cancers (Nakashima et al., 2006; Cui et al., 2010; Yamasaki et al., 2010). These findings suggest a combined transcriptional and epigenetic regulation of SHH expression. Additionally, components of the Hh pathway, such as PTCH1 and GLI1, are overexpressed in different breast cancer subtypes, with GLI1 showing particularly high expression in TNBC (Tao et al., 2011). This underscores the critical role of Hh signaling in breast cancer biology. N6-methyladenosine (m6A) methylation, an epi-transcriptomic modification, plays a crucial role in post-transcriptional regulation of RNA by modulating splicing, stability, nuclear transport, and translation efficiency. METTL3, a key methyltransferase responsible for installing m6A marks, has been implicated in various cancers (Roignant and Soller, 2017; Niu et al., 2013). Dysregulated METTL3 activity is associated with oncogenic processes, often through m6A-dependent mechanisms. In breast cancer, METTL3 inhibits the tumor-suppressive miRNA let-7g via HBXIP (Cai et al., 2018), enhances the methylation of Bcl-2 (Wang et al., 2020) to promote cell proliferation and inhibit apoptosis, and induces epithelial-mesenchymal transition (EMT) by m6A modification of EZH2 mRNA (Hu et al., 2022). Conversely, METTL3 has been shown to suppress metastasis in TNBC by modifying COL3A1, a gene involved in cell-matrix adhesion (Shi et al., 2020). These dual functions underscore the complexity of METTL3 and its context-dependent roles in breast cancer progression, reinforcing the demand for further investigations. Evidence from medulloblastoma studies indicates that METTL3 promotes SHH pathway activity by stabilizing and enhancing the translation efficiency of PTCH1 and GLI2 mRNAs (Zhang et al., 2022). Despite growing evidence linking METTL3 and SHH signaling in various cancers, the functional relationship between METTL3-mediated m6A modifications and SHH signaling in breast cancer progression remains unclear. In this study, we examined the expression levels of METTL3 and components of the SHH signaling pathway to clarify the METTL3-SHH axis in breast cancer. Our findings suggest that METTL3 overexpression promotes breast cancer progression primarily by targeting the hedgehog signaling pathway, highlighting a potential avenue for epigenetic therapy.

## 2 Methods and materials

### 2.1 Selection of study area and subjects

This research was conducted at Chittagong Medical College and Hospital (CMCH) and the Centre for Research Excellence (CRE) in the Department of Biochemistry and Molecular Biology at the University of Chittagong, Bangladesh. We prospectively recruited patients diagnosed with breast cancer for this study. A total of 35 primary tumor tissues were collected from these cancer patients, along with 20 adjacent non-cancerous tissues from the same patients to serve as controls. We managed to include a total of 55 samples in our research, with immunohistochemical grade confirmation by our collaborator from Chittagong Medical College and Hospital (CMCH). Patients were selected based on specific criteria, including having unilateral breast cancer, having undergone either mastectomy or breast-conserving surgery, the absence of other concomitant diseases, and no prior chemotherapy. The subtype of breast cancer was confirmed by physicians as part of our collaborative approach.

### 2.2 Ethical permission

This research project used a cooperative strategy for sample collection and processing. The central Bangladesh Medical Research Council (BMRC) ethics committee granted authorization for this study (approval ID 052(l) 04 06 2014). We collected samples under ethical permission approved by BMRC and Chittagong Medical College and Hospital (CMCH). Every patient and control participant provided written informed consent. The Institutional Review Board of the Chittagong Medical College and Hospital, Chittagong, Bangladesh, approved our study design and work.

### 2.3 RNA isolation and reverse transcription PCR

Total RNA was extracted from tumor samples using TRIzol reagent following the manufacturer’s guidelines. The initial RNAlater was discarded, and each tissue sample was homogenized in TRIzol while kept on ice. After centrifugation to obtain the supernatant, tissue debris was removed. Chloroform was added, and the mixture was inverted and centrifuged to achieve phase separation. The upper aqueous phase, containing the RNA, was carefully collected, and isopropyl alcohol was added to precipitate the total RNA, which was then centrifuged again. After washing and further centrifugation, the RNA pellet was dissolved in nuclease-free water. The RNA concentration was measured using a spectrophotometer (Nanodrop 2000; Thermo Scientific, United States), and purity was assessed by the 260/280 and 260/230 absorbance ratios. The GoScript™ Reverse Transcription System from Promega Corporation was used to synthesize cDNA from mRNA. The synthesis process began by isolating mRNA from total RNA using oligo (dT) primers. The mixture was heated at 70 °C for 5 min to separate the mRNA from the total RNA, followed by rapid cooling on ice for another 5 min. Next, a final volume of 20 µL was prepared by adding a specific set of reagents included in the system. This mixture was then incubated at 25 °C for 5 min, followed by incubation at 42 °C for 60 min, and finally at 70 °C for 15 min. After this process, the resulting cDNA was amplified using quantitative PCR (qPCR), where cDNA serves as the template solution.

### 2.4 Quantitative real-time PCR

The analysis of gene expression in breast cancer patients was performed using quantitative reverse transcription polymerase chain reaction (qRT-PCR) on samples taken from tumors and adjacent normal tissues. The Gapdh gene was used as a control for the study. Primers were designed using the Primer-BLAST tool from the National Center for Biotechnology Information (NCBI) (Ye et al., 2012), and all primers were provided by Integrated DNA Technologies (Table 1). Real-time PCR was done with an Applied Biosystems detection system to amplify the copy number of a target gene and detect the amplified product using SYBR Green dye as the DNA-binding fluorophore. For quantification of the genes, working cDNA samples were prepared by diluting stock cDNA to a final concentration of 50 ng/μL. Forward and reverse primers specific to the target genes were added to the diluted cDNA. SYBR Green PCR Master Mix (Promega, United States) was then added to each reaction tube, mixed thoroughly, and loaded into a real-time PCR system. The PCR reactions were carried out under the following optimized cycling conditions: one cycle at 95 °C for 2 min for heat activation, denaturation at 95 °C for 10 s, annealing at 53 °C for 10 s (adjusted based on the melting temperatures of the primers), extension at 72 °C for 30 s and repeated the process for 40 cycles. Each reaction was performed in triplicate for both the target gene and the reference gene, GAPDH, which served as the housekeeping gene for normalization. The relative changes in the expression level of a target gene were calculated using the 2^−ΔΔCt^ method.

**TABLE 1:** Primer sequences.

Gene	Direction	Sequence
SHH	Forward	5′- AGGACCCGGTTTGATCTTCT-3′
	Reverse	5′- GCCATGTGACACAGACAACC-3′
PTCH1	Forward	5′-ACAAACTCCTGGTGCAAACC-3′
	Reverse	5′- CTTTGTCGTGGACCCATTCT-3′
GLI1	Forward	5′- GTGCAAGTCAAGCCAGAACA-3′
	Reverse	5′- ATAGGGGCCTGACTGGAGAT-3′
GLI2	Forward	5′-CGAGAAACCCTACATCTGCAAGA-3′
	Reverse	5′- GTGGACCGTTTTCACATGCTT-3′
GLI3	Forward	5′- AAACCCCAATCATGGACTCAAC -3′
	Reverse	5′-TACGTGCTCCATCCATTTGGT-3′
SMO	Forward	5′- GGGAGGCTACTTCCTCATCC-3′
	Reverse	5′-GGCAGCTGAAGGTAATGAGC-3′
METTL3	Forward	5′-CCAGCACAGCTTCAGCAGTTCC-3′
	Reverse	5′-GCGTGGAGATGGCAAGACAGATG-3′
GAPDH	Forward	5′- CAGCCTCAAGATCATCAGCA -3′
	Reverse	5′- TGTGGTCATGAGTCCTTCCA- 3′

### 2.5 Bioinformatics analysis

Bioinformatics analyses were performed utilizing data from multiple publicly available platforms, including UALCAN (https://ualcan.path.uab.edu, updated on: 2022, accessed on 10 December 2024), cBioPortal (https://www.cbioportal.org/api/info, version: v6.3, accessed on 18 December 2024), and GEPIA (Gene Expression Profiling Interactive Analysis, http://gepia.cancer-pku.cn/index.html, accessed on 9 December 2024), to comprehensively investigate the methylation patterns, gene expression levels, and mutation profiles of specific genes of interest. UALCAN served as a valuable resource for examining differential methylation across various cancer types, while cBioPortal offered insights into the genetic alterations and mutation frequencies within specific cohorts. GEPIA contributed to assessing the expression profiles of the selected genes, enabling a comparative analysis between tumor and normal tissue samples.

### 2.6 Statistical analysis

A two-tailed Student’s t-test was performed for statistical analysis using GraphPad Prism 8.5.2. The Spearman and Pearson coefficient (r) test was performed using the same statistical package for correlation analysis between two genes. Comparisons between groups were performed using a one-way ANOVA test. Survival analysis was performed using the Kaplan–Meier (KM) method for patients’ outcomes based on either overall survival or disease-free survival. Hazard ratio (HR) with 95% confidence intervals was calculated to estimate the relative risk associated with gene expression, HR = 1: no difference; HR > 1: increased risk; HR < 1: reduced risk, and statistical significance of survival curves for high versus low groups is evaluated using the log-rank test (p < 0.05). Data were expressed as mean ± standard deviation, and a p-value of less than 0.05 was considered statistically significant. *p < 0.05, **p < 0.01, ***p < 0.001.

## 3 Result

### 3.1 METTL3 overexpression correlates with poor survival in breast cancer patients

**FIGURE 1:**
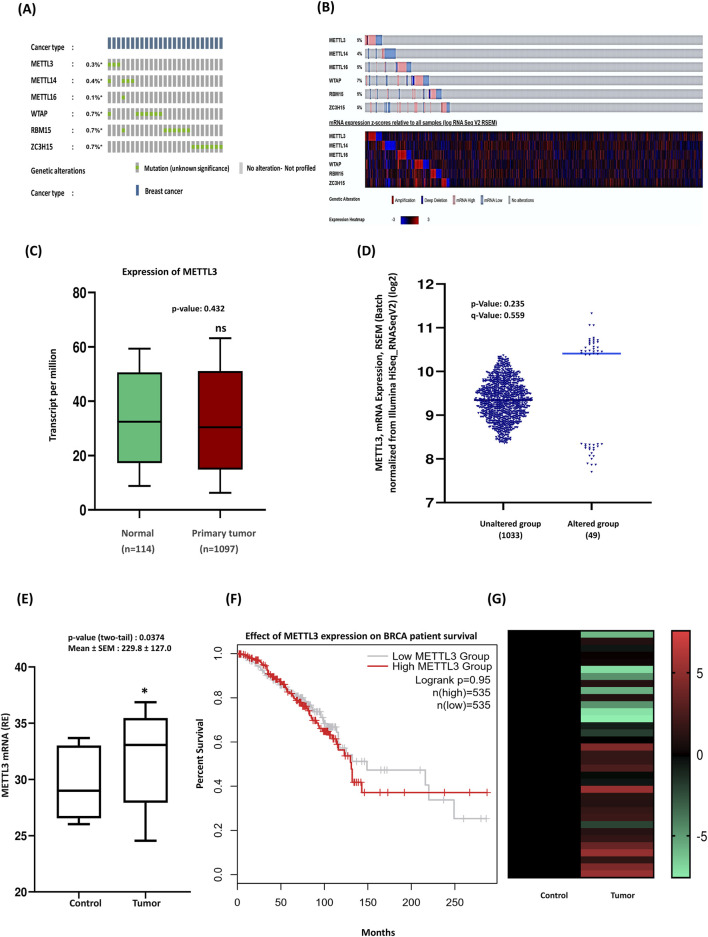
Upregulation of METTL3 expression and its implications in breast cancer patients’ outcomes. **(A)** Oncoprint data summarizing the genomic alterations of methyltransferase complex genes (METTL3, METTL14, METTL16, WTAP, RBM15, and ZC3H15) in 1084 TCGA samples. Colored sections represent mutations, with moderate alterations observed in METTL3. Queried genes are altered in 2% of patients. **(B)** The heatmap provides a detailed view of gene expression (low to high) in individual samples, were altered in 242 (22%) of 1,084 samples. **(C)** Boxplot of METTL3 expression in tumor and normal samples. **(D)** cBioPortal analysis highlights significant expression differences between patients with METTL3 alterations and those without. **(E)** qRT-PCR of METTL3 expression in clinical samples (n = 35) compared to normal tissues. **(F)** Kaplan-Meier survival curve: patients are divided into two groups based on METTL3 expression levels: The low METTL3 expression group (black line) and the High METTL3 expression group (red line). **(G)** Experimental validation of METTL3 expression trends across clinical samples in a heatmap. For comparison between healthy and cancer cases, two-tailed unpaired t-tests were performed in GraphPad Prism, with significance determined at p < 0.05. Error bars represent the mean ± S.D. of the tissue samples. *p < 0.05 vs. control.

Many types of human cancers exhibit fluctuating RNA m6A modifications, primarily due to dysfunctional m6A regulators, which include writers, erasers, and readers (Huang et al., 2020). Dysregulated RNA m6A methylation can result in the activation of oncogenes or the inhibition of tumor suppressor genes by targeting specific signaling pathways involved in cancer. Research has shown that imbalanced RNA m6A methylation, caused by METTL3 in the cerebellum, results in defective cell proliferation and differentiation of granular neuronal cells. Among the various m6A regulators identified to date, METTL3 is a core component of the methyltransferase complex and is overexpressed in all cases of medulloblastoma (Weishaupt et al., 2019). To explore the impact of m6A methylation by METTL3 on breast cancer tumorigenesis, we first analyzed the molecular alterations of METTL3 compared to other components of the methyltransferase complex. We found that the mutation frequencies of METTL3 among the m6A writers were low across all subunits (Figure 1A). Next, we assessed the mRNA expression levels and observed aberrant expression patterns of methyltransferase complex genes (Figure 1B). Specifically, our analysis of METTL3 expression in breast cancer using The Cancer Genome Atlas (TCGA) dataset revealed decreased METTL3 levels in breast cancer tissues compared to healthy individuals (Figure 1C). However, this reduction was not statistically significant enough (p > 0.05) to conclude that METTL3 is downregulated in breast cancer. The expression of METTL3 in different BC subtypes (Luminal, HER2 positive, and TNBC), based on molecular heterogeneity, was investigated to determine if METTL3 expression correlated with BC subtypes. Analysis of METTL3 mRNA expression via cBioPortal highlights significant differences between breast cancer samples with and without METTL3 gene alterations. Approximately 5% of breast cancer samples exhibit alterations in the gene. Notably, the altered group shows higher mRNA expression than the unaltered group, suggesting that alterations may be associated with increased expression levels of METTL3 (Figure 1D). qRT-PCR analysis of 35 breast cancer samples of our patient cohort from Bangladesh, distinct from the TCGA dataset, revealed higher METTL3 expression compared to healthy tissues (Figure 1E). This apparent discrepancy may arise from differences in cohort composition, sample size, and tumor heterogeneity. We also analyzed the expression of METTL3 in subtype-specific breast cancers in the TCGA database (Supplementary Figure S1A) and found downregulation in TNBC and Luminal subtypes, while the HER2-positive subtypes expressed a slightly higher amount of METTL3. The analysis of three subtypes of BC by qRT-PCR revealed upregulation of METTL3 in luminal and HER2-positive subtypes, but not statistically significant (P > 0.05), whereas it was significantly overexpressed in the TNBC subtype (Supplementary Figure S1B). These findings suggest that bulk TCGA data may dilute subtype or region-specific patterns, while cohort-based studies in Bangladesh highlight context-dependent differences in METTL3 expression that are clinically relevant for local patients. However, METTL3 expression does not appear to significantly impact the survival outcomes of BRCA patients in the TCGA cohort (Figure 1F). Additionally, we evaluated the differential expression of METTL3, noting that the control group consistently exhibited lower expression levels of METTL3 (dark shades), whereas tumor samples had elevated expression (lighter shades) (Figure 1G). This suggests the upregulation of METTL3 in tumor tissues, potentially linked to its role in tumor development and progression. Across public databases, METTL3 expression patterns were inconsistent, but our qRT-PCR validated significant upregulation in our patient cohort. Previous studies have shown that advanced breast cancer patients, particularly those with lymphatic metastasis or poor prognosis, often have elevated METTL3 levels, emphasizing its importance in breast cancer progression (Xie et al., 2021). Research by Xie et al. highlighted the role of METTL3 in promoting breast cancer stemness, demonstrating that upregulated METTL3 enhances both stemness and malignant progression by mediating m6A modifications to SOX2 mRNA (Xie et al., 2021). Survival curves of the TCGA cohort by molecular subtype indicated that high METTL3 expression correlates with poor survival in TNBC (data not shown here), likely reflecting subtype-specific effects that are diluted in bulk analyses. Current literature and public datasets suggest that METTL3 levels may be closely linked to disease progression. Overall, our findings suggest that METTL3 may act as an oncogenic driver, modifying mRNAs and triggering the onset and progression of breast cancer.

### 3.2 Role of sonic hedgehog in breast cancer progression

**FIGURE 2:**
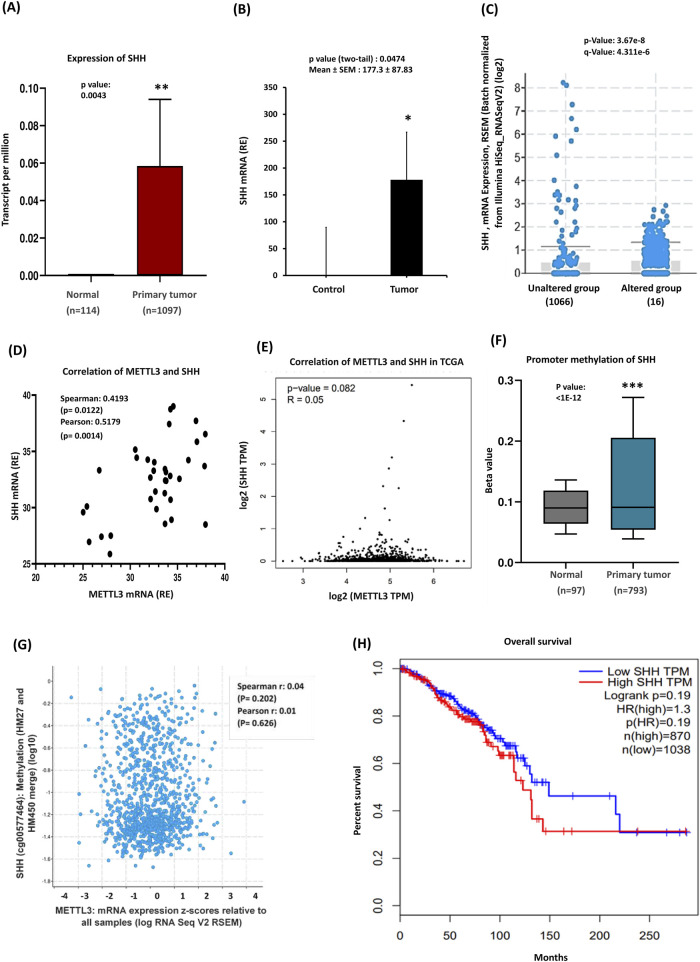
Sonic Hedgehog expression, methylation, and clinical significance in breast cancer. **(A)** TCGA data highlights SHH overexpression in breast cancer (BRCA). **(B)** qRT-PCR of SHH mRNA levels, which are significantly elevated in tumor tissues (n = 35) compared to controls. **(C)** 1% of Patients exhibited alterations in their mRNA expression, with the altered group showing higher expression of certain mRNAs than the unaltered group. **(D)** Correlation between METTL3 and SHH expression, Pearson and Spearman analyses were conducted in GraphPad Prism. **(E)** GEPIA analysis further explores the relationship between METTL3 and SHH. **(F)** Promoter methylation analysis: the Beta value indicates the level of DNA methylation, ranging from 0 (unmethylated) to 1 (fully methylated). Different beta value cut-offs have been considered to indicate hypermethylation [Beta value: 0.7–0.5] or hypomethylation [Beta value: 0.3–0.25]. **(G)** A scatter plot illustrating the relationship between SHH methylation and METTL3. **(H)** Survival analysis of breast cancer patients based on the Kaplan–Meier plotter database. p-values were calculated using a two-tailed Student’s t-test, with p < 0.05 considered statistically significant; ns = non-significant (p > 0.05). ***p < 0.001 vs. control.

Recent studies suggest that aberrant activation of the hedgehog cascade by elevated SHH ligands plays a significant role in driving tumor growth in human breast cancer (Mukherjee et al., 2006; Kubo et al., 2004). To investigate the regulatory role of the SHH pathway and its associated genes in breast cancer, we performed real-time PCR on breast cancer tissue samples. According to TCGA, elevated SHH expression had been observed in all subtypes of BC (Supplementary Figure S2A). Analysis of the UALCAN database, which included 1,097 breast-invasive carcinoma samples, further confirmed a higher pattern of SHH expression in primary tumors (Figure 2A). We next assessed SHH expression in breast carcinomas compared to normal breast tissues through quantitative RT-PCR analysis. In normal breast tissues, SHH expression was low, whereas tumor tissues exhibited significantly higher SHH expression (P < 0.05) (Figure 2B), validating findings from the public database. Subtype-specific analysis revealed notable differences in SHH expression compared to the dataset, particularly in luminal breast cancer, where triple-negative breast cancer and HER2-positive subtypes showed similar, but not as pronounced as in luminal BC (Supplementary Figure S2B). In a study examining mRNA expression levels, it was found that 1% of patients exhibited alterations in their mRNA expression compared to a control group that showed no such changes (Figure 2C). Moreover, the altered group displayed higher expression of certain mRNAs than the unaltered group, suggesting a link to particular biological processes or pathways relevant to the patients’ conditions. Our experimental findings indicated a role for METTL3 in breast tumors, prompting us to investigate the relationship between these two genes. A scatterplot depicted the correlation between METTL3 and SHH mRNA expression levels, with both Spearman and Pearson correlation analyses revealing a positive relationship (Figure 2D). This suggests that higher METTL3 expression is associated with elevated SHH levels. To further confirm the role of METTL3, we examined the methylation patterns of SHH and its correlation with METTL3 by using various public databases. GEPIA analysis indicated a weak relationship between METTL3 and SHH (Figure 2E). The methylation patterns of SHH (cg00577464) CpG sites (HM47 and HM450) within the proximal promoter regions showed minimal correlation with METTL3 expression (Figure 2F). The scatter plot depicting SHH methylation versus METTL3 expression illustrated a weak correlation as indicated by low Spearman and Pearson values (Figure 2G). The data points were widely scattered, showing no discernible trend, suggesting that variations in METTL3 expression do not significantly affect SHH methylation. However, the database indicated significant differences in SHH promoter methylation between normal and tumor tissues, suggesting potential regulatory mechanisms. Generally, promoter methylation represses gene expression; yet in some cancers, hypermethylation can increase expression through its regulatory effects on chromatin stability. Considering our findings, we speculated that high promoter methylation in tumors may stabilize SHH mRNA or regulate its transcription differently in breast cancer, although this requires experimental validation. Further studies are needed to clarify METTL3’s impacts on mRNA stability or transcriptional efficiency. Our qRT-PCR results demonstrate SHH overexpression in tumors, aligning with our methylation and correlation studies and reinforcing SHH’s oncogenic role in breast cancer. Our data are consistent with the idea that METTL3 enhances SHH expression through m6A-dependent stabilization, driving tumor progression, although they require further experimental validation of METTL3 overexpression. Additionally, Kaplan-Meier survival curves illustrate the impact of SHH expression on overall patient survival, emphasizing its complex role in prognosis (Figure 2H).

### 3.3 Expression of transmembrane protein PTCH1 in breast cancer

**FIGURE 3:**
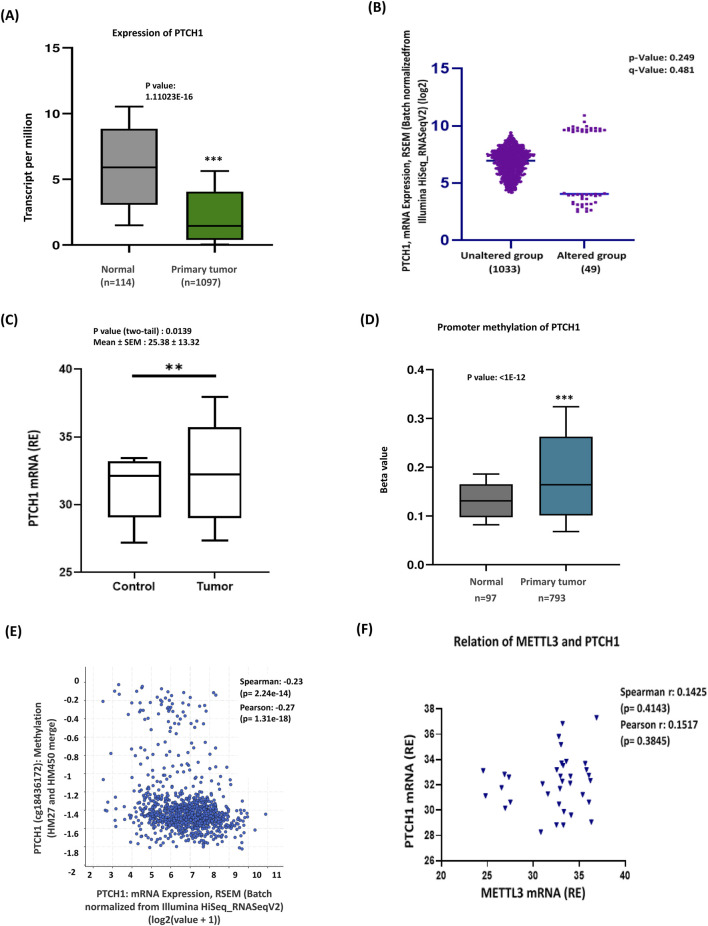
Promoter hypermethylation reduced PTCH1 expression in breast cancer. **(A)** TCGA database analysis shows a significantly lower expression of PTCH1 in breast cancer tissues. **(B)** The cBioportal analysis of PTCH1 revealed a 5% alteration in expression, with some regions affected, while the surrounding unaltered areas exhibit a robust expression profile. **(C)** Experimental data showing increased PTCH1 expression in breast cancer (n = 35) compared to control tissues. **(D)** Methylation analysis supporting PTCH1 promoter hypermethylation in primary tumors. The gene in question is altered in 49 (5%) of the queried patients or samples. **(E)** Correlation of PTCH1 methylation and mRNA expression (Spearman: 0.23, Pearson: 0.27). This negative correlation suggests that hypomethylation leads to PTCH1 activation. **(F)** Correlation between PTCH1 mRNA and METTL3 mRNA. ***p < 0.001 vs. control.

The Hedgehog (Hh) signaling pathway is essential for developmental processes and the regulation of cancer stem cells. This pathway is initiated when Hh ligands attach to the Patched1 (PTCH1) protein (Cierpikowski et al., 2023). This interaction causes the removal of PTCH1’s suppression on the Smoothened protein (SMO), ultimately activating the GLI complex and leading to the transcription of target genes. PTCH1 functions as a negative regulator of the Hh signaling pathway, and mutations that impair its function can lead to inappropriate activation of this pathway (Patel et al., 2023; Covarrubias-Pinto et al., 2022). In contrast, SMO acts as a positive regulator, as it becomes active when PTCH1’s inhibitory effect is absent (Skoda et al., 2018). Given this information, next, we aimed to examine the expression of the Hh pathway activators—PTCH1 and SMO—and their correlation with METTL3 to explore the epigenetic influence of METTL3 on Hh signaling activation. The TCGA database revealed a significant decrease in the mRNA expression levels of PTCH1 in breast cancer tissues compared to normal tissues (Figure 3A). Recent analyses using data from cBioPortal have revealed intriguing insights regarding PTCH1 mRNA alterations in breast cancer patients. Subtype-specific analysis indicated PTCH1 expression decreased significantly in luminal, HER2+, and TNBC (Supplementary Figure S3A). The findings indicate that there is a low prevalence of mRNA alterations within this patient cohort. Interestingly, this analysis also suggests that the group of patients without these alterations exhibits a higher overall expression of PTCH1 mRNA. This could imply that, despite the lower frequency of mRNA changes, those breast cancer patients without alterations may have different biological profiles or responses to treatment (Figure 3B). qRT-PCR data of our patient cohort corroborated these findings, demonstrating a notable increase in expression of PTCH1 in breast cancer tissues compared to adjacent normal tissues (Figure 3C; Supplementary Figure S3B). We further investigated the methylation status of the PTCH1 gene, which was depicted in a bar diagram showing hypomethylation of the PTCH1 promoter (Figure 3D). This decrease in methylation is implicated in the activation of PTCH1. Furthermore, bioinformatic analysis identified a negative correlation between PTCH1 mRNA methylation and gene expression, providing additional support for these conclusions (Figure 3E). The experimental data showed a positive but not statistically significant relationship between METTL3 and PTCH1 mRNA levels (Figure 3F), reflecting high variability in expression with no clear trend.

### 3.4 Relationship among PTCH1, SMO, and METTL3 expressions in breast cancer

**FIGURE 4:**
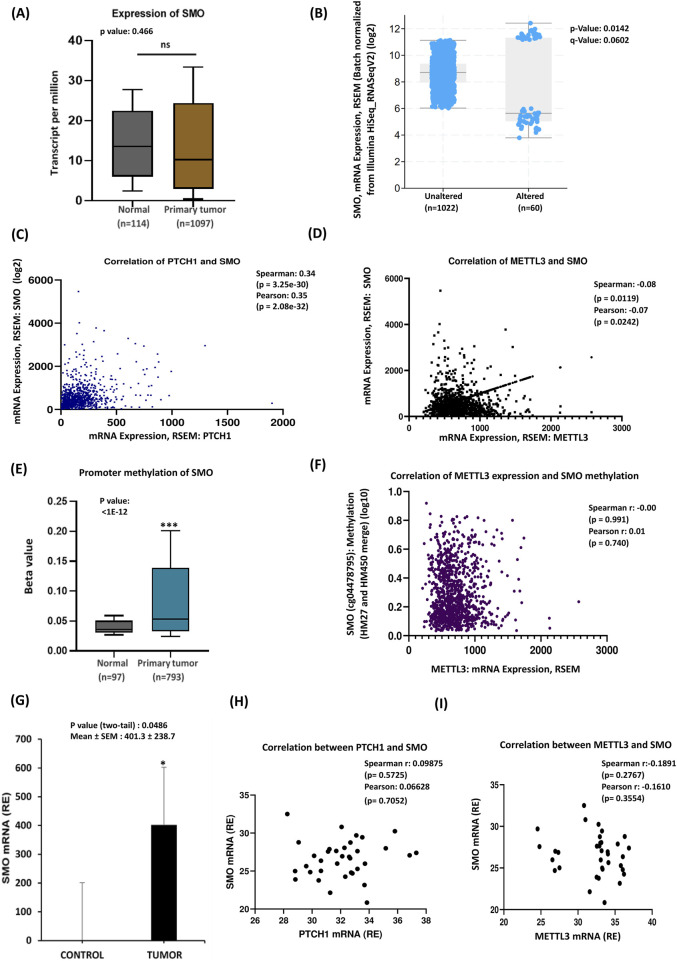
SMO mRNA alteration is associated with PTCH1 and METTL3 expressions. **(A)** TCGA data analysis of breast cancer patients. **(B)** SMO mRNA expression alteration: the graph compares SMO mRNA expression between the altered and unaltered groups. **(C)** Co-expression analysis of PTCH1 and SMO. **(D)** Co-expression of METTL3 and SMO: the scatterplot shows a positive correlation between METTL3 and SMO mRNA. **(E)** Box plot compares SMO promoter methylation levels between normal tissue (n = 97) and primary tumor tissue (n = 793). **(F)** The relationship between METTL3 mRNA expression (x-axis) and SMO methylation levels (y-axis). **(G)** Experimental validation of SMO mRNA overexpression in tumor samples (n = 35) compared to control samples. **(H)** Correlation analysis between PTCH1 and SMO. **(I)** Co-expression between METTL3 and SMO expression. *p < 0.05 vs. control.

As the binding of SHH to its receptor PTCH1 releases its inhibition of smoothened (SMO), allowing it to become active and initiate the downstream signaling cascade, we intended to investigate the expression of SMO and its relationship with PTCH1 in our patient cohort. Analysis of the TCGA database on breast cancer patients revealed a significant reduction in SMO gene expression levels in tumor samples compared to healthy tissue (Figure 4A), however, subtype-specific analysis revealed increased expression of SMO in TNBC (Supplementary Figure S4A). The transmembrane protein smoothened was altered in approximately 6% (60 samples/patients) of breast cancer cases, with higher expression levels found in the unaltered group (Figure 4B). Numerous studies have established the involvement of PTCH1 and SMO in oral cancer (Patel et al., 2024). Additionally, increased PTCH1 protein expression has been observed in various malignancies, including breast, prostate, lung, colon, and brain cancers (Wang et al., 2019; Chung and Bunz, 2013). Furthermore, mutations in SMO expression are often associated with medulloblastoma and basal cell carcinomas (Dhanyamraju et al., 2020). Thus, we next checked the correlation between PTCH1 and SMO expressions, where the scatterplot indicated a moderate positive correlation showing an upward trend in SMO expression with increasing PTCH1 levels (Figure 4C). This suggests that samples with higher PTCH1 mRNA levels tend to exhibit elevated SMO expression. In the analysis of METTL3 and SMO correlation, the scatterplot showed a trend where higher METTL3 mRNA expression may be associated with lower SMO mRNA expression, although this relationship is statistically significant (Figure 4D). Comparison of SMO promoter methylation levels between normal tissue (n = 97) and primary tumor tissue (n = 793), demonstrating significantly higher methylation levels in primary tumors (Figure 4E). This finding suggests SMO methylation may play a role in tumorigenesis. Further examination of the relationship between METTL3 mRNA expression levels and SMO methylation levels illustrated no significant correlation (Figure 4F), indicating that variations in METTL3 mRNA may not significantly affect SMO methylation. This lack of correlation suggests distinct regulatory mechanisms, highlighting the need for further investigation into the roles of METTL3 and SMO in biological processes and disease pathways. In contrast to database findings, experimental data of SMO showed that significantly elevated SMO mRNA expression in tumors compared to the control group (Figure 4G), suggesting that upregulated SMO in breast cancer tissues may drive tumor cell proliferation and progression. Subtype-specific analysis indicated significant upregulation of SMO in luminal and HER2 subtypes but not in TNBC (Supplementary Figure S4B). The experimental analysis of the correlation between PTCH1 and SMO revealed a positive albeit not statistically significant relationship, as indicated by both Spearman and Pearson statistical tests. Furthermore, regression analysis indicated a slight positive slope of the regression line, suggesting a possible interaction between these two genes (Figure 4H). Additionally, we analyzed the correlation between METTL3 and SMO mRNA levels from qRT-PCR data in breast cancer. The Spearman and Pearson correlation coefficients (−0.1891 and −0.1610, respectively) indicate statistically nonsignificant negative relationships (Figure 4I). Although bioinformatics analysis has identified a statistically significant, albeit weak, negative correlation between METTL3, SMO, and PTCH1, experimental data did not corroborate these findings. Based on our analysis, we suggest that the heightened expression of both PTCH1 and SMO may have a role in activating Hedgehog (Hh) signaling. Moreover, METTL3 may play an indirect regulatory role in Hh signaling by influencing the stability or translation of upstream activators. To clarify these relationships and mechanisms further, additional experimental studies are necessary.

### 3.5 Role of GLI1, GLI2 and GLI3 molecules in breast cancer progression

**FIGURE 5:**
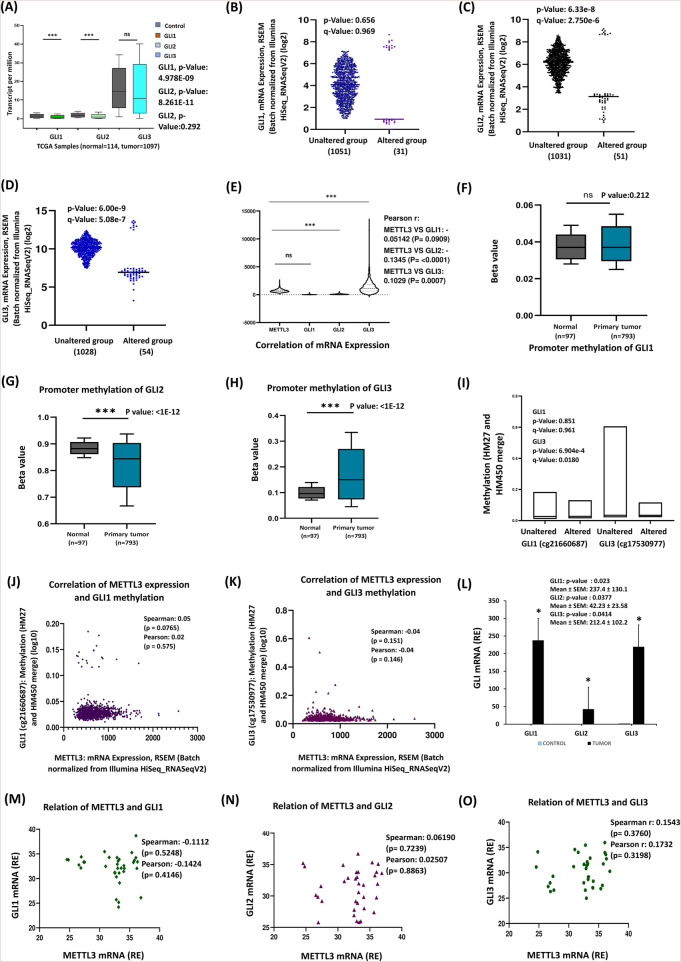
Expression and methylation pattern of GLI transcription factors: **(A)** Patterns of expression for the GLI family of genes—GLI1, GLI2, and GLI3. **(B–D)** The analysis of mRNA expression from cBioPortal. **(E)** Correlation of mRNA expression levels between the METTL3 and GLI family genes. **(F–H)** The boxplot compares promoter methylation of GLI, GLI2 and GLI3 genes in normal and primary breast cancer tissues. **(I)** Methylation patterns of GLI1 and GLI3. GLI2 does not show methylation data in the database. **(J,K)** The data represent the relationship between METTL3 expression and the methylation patterns of the GLI1 and GLI3 genes. **(L)** GLI1, GLI2, and GLI3 mRNA expression levels are measured in tumor (n = 35) and control samples by qRT-PCR. **(M–O)** The scatter plot showing the correlation of METTL3 and GLI genes in experimental data. ***p < 0.001 vs. control.

Our study demonstrates that tumor tissue exhibits higher expression levels of SHH, PTCH1, and SMO mRNAs compared to noncancerous tissue, correlating with breast cancer progression. Experimental data, literature findings, and bioinformatics analyses suggest that in breast cancer, the SHH ligand binds to activate PTCH1 and SMO, indicating a possible role for downstream molecules. This prompted our analysis of the genes involved in tumorigenesis and breast cancer progression. The glioma-associated oncogene homologs, GLI1, GLI2, and GLI3, are closely regulated by Hedgehog (Hh) signaling. Notably, GLI1 serves as a key target gene in the SHH pathway and positively correlates with SHH in breast tissues (Bale and ping, 2001; Johnson et al., 1979). Furthermore, GLI1 is crucial for the proliferation, survival, and migration of inflammatory breast cancer (Thomas et al., 2011). Therefore, in our study, we focused on the comparative gene expression of three specific genes associated with cancer progression. Analysis of the database revealed distinct expression patterns of the GLI gene family—GLI1, GLI2, and GLI3—in tumor versus control samples (Figure 5A). GLI1 and GLI2 were significantly downregulated in tumors (p < 0.001), suggesting they act as negative regulators in cancer. In contrast, GLI3 showed no significant changes, indicating it may not be directly involved in tumorigenesis. These results underscore the differential regulation of GLI genes in cancer and merit further investigation into their roles in tumor development. cBioPortal data revealed that some patients exhibited alterations in the GLI family of genes (Figures 5B–D). Notably, the GLI1 alterations were observed in 3% of the patients, while both GLI2 and GLI3 had higher alteration frequencies at 5% each. The expression in the altered group was significantly lower compared to the unaltered group. Additionally, mRNA expression levels of METTL3, GLI1, GLI2, and GLI3 were batch-normalized from RNA sequencing results (Figure 5E), revealing a significantly higher expression pattern for the GLI2 and GLI3 genes, suggesting a strong possible relationship with METTL3. This correlation may suggest that m6A mRNA modifications serve as regulatory mechanisms for these genes. In contrast, the GLI1 gene exhibited no significant correlation with METTL3 expression. Promoter methylation analysis revealed elevated levels of promoter methylation for GLI3 and decreased methylation for GLI2 in tumors, while GLI1 did not exhibit prominent changes (Figures 5F–H). Methylation pattern analysis derived from the public dataset scrutinized the CpG sites for GLI1 and GLI3 and indicated no significant changes for GLI1 between altered and unaltered groups (p = 0.851), but significant alterations for GLI3 with a p-value of 6.904e^-6 (Figure 5I). The findings of correlation analyses suggested that there may be a positive correlation between METTL3 expression and GLI1 methylation, whereas a possible weak negative correlation between METTL3 expression and GLI3 methylation, though these relationships were not statistically significant (Figures 5J,K). Our experimental findings showed that all three GLI genes exhibited significant upregulation in expression when comparing cancerous tissues to noncancerous counterparts (Figure 5L), contradicting the TCGA cohort. Notably, GLI1 demonstrated the highest expression, indicating it may have a prominent role in this context, followed by GLI3. This differential expression highlights the potential importance of these genes, particularly GLI1 and GLI3, in understanding the molecular mechanisms underpinning cancer development. Studies on gene expression patterns in breast cancer have emphasized significant variations among different subtypes. Notably, the expression levels of GLI1, GLI2, and GLI3 genes exhibited distinct patterns tied to these subtypes, which are crucial for understanding tumor behavior and guiding treatment strategies (Supplementary Figures S5A–F). GLI1 showed a pronounced upregulation in the HER2 subtype in experimental data but was downregulated across all subtypes in the TCGA database (Supplementary Figures S5A, B). GLI2 showed high expression across all subtypes in qRT-PCR analysis, whereas it has low expression in all subtypes of breast cancer in TCGA (Supplementary Figures S5C, D). Conversely, GLI3 is upregulated primarily in the Luminal subtype and downregulated in HER2 and TNBC in the TCGA database but upregulated in all subtypes in qRT-PCR data (Supplementary Figures S5E, F), suggesting GLI3 may be involved in the biological processes associated with Luminal breast cancers, which are known for their distinct hormonal characteristics. Differences in observed expression trends across public databases may be influenced by factors such as subtype composition, sample size, and tumor heterogeneity. qRT-PCR analysis indicated the expression of GLI family genes may align closely with that of METTL3 expression (Figures 5M–O). There is a weak positive correlation between METTL3 mRNA expression with GLI2 and GLI3 genes, while GLI1 showed a negative correlation. These findings suggest a probable potential interaction or shared regulatory mechanism between METTL3 and the GLI family genes, highlighting the need for further investigation into their roles in tumor biology.

## 4 Discussion

The stemness and progression of breast cancer involve the complex interplay of various genes and signalling pathways. This research aimed to investigate the influence of METTL3 on BC tumorigenesis and progression through the SHH signalling pathway, revealing significant insights into their roles across all BC subtypes. Our research has uncovered a significant upregulation of several critical factors associated with tumor progression in breast cancer. Specifically, we observed elevated levels of METTL3, the SHH ligand, and pivotal components of the SHH signaling pathway, including PTCH1, SMO, GLI1, GLI2, and GLI3. Notably, these upregulations were observed across all analysed breast cancer subtypes in our cohort. The TCGA vs. cohort discrepancies can be demonstrated by tumor purity, subtype composition, and data-processing methods. Furthermore, our analysis revealed a positive correlation between METTL3 and SHH, indicating a possible potential functional interplay between these two. This relationship suggests that METTL3 might facilitate or amplify SHH signalling, possibly through mechanisms like post-transcriptional modifications, such as N6-methyladenosine (m6A) methylation.

The process of m6A modification is regulated by various proteins and enzymes, such as m6A-binding proteins, demethylases, and methyltransferases (Fu et al., 2021). m6A methyltransferase METTL3 plays a crucial role in tumor progression by catalyzing m6A modifications of adenosine on mRNA (Lan et al., 2021). These m6A modifications influence mRNA at multiple levels, including splicing, stability, structure, translation, export, decay, and maturation (Liu et al., 2018). Its increased expression in breast cancer suggests a role in enhancing the expression of oncogenic mRNAs, thereby promoting tumor growth and survival. By altering transcripts involved in cell proliferation and differentiation, METTL3 may contribute to the aggressive behavior seen in breast cancer cells (Wang et al., 2020).

SHH is a secreted ligand that initiates the SHH signaling pathway, which is essential for embryonic development and tissue patterning. In breast cancer, overexpression of Shh has been associated with tumor progression and poor prognosis. Upregulation of SHH can activate downstream signaling components, leading to cellular proliferation and contributing to the malignancy of breast cancer (Cui et al., 2010; Kuehn et al., 2021). The role of METTL3 in cancer is increasingly recognized, particularly for its ability to regulate m6A modifications of transcripts involved in oncogenesis (Wang et al., 2020). In breast cancer, METTL3 may influence the stability or translation efficiency of SHH-related transcripts, thereby amplifying the SHH signaling cascade (Cai et al., 2019). This idea aligns with prior studies showing that METTL3-mediated m6A modification stabilizes oncogenic mRNAs, promoting tumor growth and invasion. Our research provides strong evidence for the oncogenic role of the Sonic Hedgehog (SHH) signaling pathway in cancer progression. Notably, we found that both METTL3 and SHH are significantly upregulated in cancer patients (Figures 1, 2), suggesting they may be involved in tumor development and growth. This increase suggests a likely interaction between these molecules, which may contribute to the aggressive nature of the malignancy. SHH signaling components (PTCH1, SMO, and GLI family) are known to drive proliferation, survival, and stemness in cancer cells, but the specific mechanisms governing the regulation of SHH mRNA itself remain unclear. This gap in understanding highlights an important area for further research, as direct modifications to Shh mRNA could potentially alter the expression and activity of downstream signaling components, thereby influencing tumor dynamics.

Interestingly, the consistent upregulation of METTL3 and Shh signaling components across all breast cancer subtypes highlights their universal importance in the disease. While previous studies have reported subtype-specific differences in the expression of various oncogenic pathways, our data suggest that the METTL3-SHH axis might serve as a common driver of malignancy, regardless of the molecular subtype. Beyond directly regulating Shh transcripts, METTL3 may influence other pathway components or interact with additional epigenetic regulators, further boosting the oncogenic potential of SHH signaling. This layered regulation requires further investigations to understand the exact molecular mechanisms linking METTL3 and SHH in breast cancer (Cai et al., 2019). Patched homolog 1 (PTCH1) is an important membrane receptor that is involved in the Hedgehog (Hh) signaling pathway and linked to cancers, including lung, prostate, ovarian, melanoma, brain, myeloid leukemia, breast, and colon cancers (Hasanovic and Mus-Veteau, 2018). Studies have shown that colorectal cancer tissues often exhibit increased expression of PTCH1. In breast cancer, this upregulation may indicate an adaptive response to elevated Sonic Hedgehog (SHH) levels, reflecting an increase in pathway activity and suggesting the existence of a feedback mechanism within the tumor microenvironment (Papadopoulos et al., 2016). Liang et al. reported findings on esophageal cancer cell lines, demonstrating that METTL3 is highly expressed and enhances PTCH1 stability by increasing its m6A modification (Liang et al., 2023). In our study, we observed that PTCH1 is upregulated; however, it demonstrates only a weak correlation with METTL3, suggesting that PTCH1 may be regulated by METTL3 in the context of breast cancer progression.

Smoothened (SMO) is a transmembrane protein that transduces signals in the Hedgehog (Hh) pathway following the inhibition of Patched 1 (PTCH1) (Chai et al., 2021). Its overexpression in breast cancer tissues indicates active Hh signaling. Research by Patel et al. has underscored the significance of PTCH1 and SMO in the progression and advancement of oral cancer, establishing their potential involvement at both the protein and mRNA levels (Patel et al., 2024). Further evidence of abnormal Hh pathway activity includes PTCH1 and SMO mutations identified in sporadic basal cell carcinomas and medulloblastomas (Patel et al., 2024). In our clinical samples, SMO transcript levels were notably upregulated, paralleling an increase in METTL3 expression, suggesting that METTL3 may facilitate SMO overexpression, signifying active Hh signaling in breast cancer. Typically, SMO is localized on the cell membrane and activated by Hh ligand binding to the PTCH1 receptor; however, its localization in the cytoplasm may indicate disrupted Hh signaling pathway activity (Patel et al., 2023; Patel et al., 2024).

GLI transcription factors (GLI1, GLI2, and GLI3) are the principal effectors of the Sonic Hedgehog (SHH) pathway, crucial for regulating gene expression associated with cell cycle progression and survival. In breast cancer, increased expression of these factors is linked to heightened transcriptional activity of oncogenic targets, contributing to tumor growth and poor clinical outcomes. Zhang et al., demonstrated that SHH-Medulloblastoma (SHH-MB) showed significant RNA m6A hypermethylation compared to normal cerebella, with overexpression of the m6A methyltransferase METTL3 leading to hypermethylation of PTCH1 and GLI2. This hypermethylation promotes sustained hedgehog signaling, further advancing SHH-MB tumor progression (Zhang et al., 2022). Additionally, in prostate cancer, METTL3 depletion resulted in decreased GLI1 expression and significantly reduced mRNA levels of downstream SHH signaling targets, such as c-Myc and Cyclin D1, ultimately inducing cell apoptosis (Cai et al., 2019). Notably, elevated levels of GLI1, GLI2, and GLI3 correlate with worse relapse-free survival in breast cancer patients, particularly in HER2-positive tumors (Zhang et al., 2022; Mastrangelo and Milani, 2018). While previous studies showed no significant changes in the relationship between METTL3 and GLI3, our study identified a noteworthy connection between them, suggesting potential interactions that warrant further exploration. The increase in GLI1, GLI2, and GLI3 levels in our findings indicates that METTL3 may enhance SHH signaling, activating the transcription of downstream target genes and contributing to tumorigenesis.

The upregulation of METTL3, along with components of the SHH signaling pathway, suggests a potential synergistic effect in promoting breast cancer progression. METTL3-mediated m6A modifications may enhance the stability and translation of mRNAs encoding SHH pathway proteins, thereby amplifying the signaling output and creating a feed-forward loop that exacerbates tumorigenic processes. This interplay emphasizes the importance of considering both epigenetic modifications and signaling pathways when developing therapeutic strategies. The overexpression of METTL3 and SHH pathway components presents viable targets for intervention; inhibitors of METTL3 could disrupt m6A-mediated stabilization of oncogenic transcripts, while targeting SHH pathway components, such as SMO or GLI transcription factors, may impede aberrant signaling. Combining these approaches could form a comprehensive strategy to attenuate breast cancer progression, particularly in subtypes exhibiting pronounced upregulation of these genes. The therapeutic implications of our findings are promising, as pharmacological inhibition of METTL3 or key components of the SHH pathway may provide novel treatment options. Given the widespread upregulation of METTL3 and SHH signaling across all breast cancer subtypes, such targeted therapies may effectively address the heterogeneity of the disease. However, this study has several limitations, our patient cohort was relatively small and from a single, which may restrict the generalizability of the results. In addition, pre-analytical variability in tissue processing and RNA quality could have influenced expression measurements. Reliance on public datasets introduces further challenges related to platform heterogeneity and batch effects. Finally, we could not perform functional validation experiments (METTL3 knockdown/overexpression) to establish the mechanistic role of METTL3. The study demonstrates primarily correlative evidence and future studies incorporating larger, multi-center cohorts and experimental validation are necessary to confirm the prognostic and therapeutic relevance of METTL3.

## 5 Conclusion

In our study, we demonstrated novel findings that indicate the m6A methyltransferase METTL3 is significantly upregulated in breast cancer tissues. This increased expression of METTL3 plays a critical role in tumorigenesis and breast cancer progression. Furthermore, we elucidate that METTL3 activates the Sonic Hedgehog (SHH) signaling pathway, which impacts downstream targets contributing to breast cancer progression. We demonstrated a strong possible correlation between METTL3 and SHH suggesting that METTL3 may serve as a crucial upstream regulator of SHH signaling. This highlights the potential of both METTL3 and SHH as universal therapeutic targets and underscores the need for further research incorporating mechanistic experiments to clarify the specific mechanisms by which METTL3 regulates SHH signaling for clinical applications.

## Data Availability

The original contributions presented in the study are included in the article/Supplementary Material, further inquiries can be directed to the corresponding author.

## Abbreviations

BC, Breast cancer; BMRC, Bangladesh Medical Research Council; CRE, Centre for Research Excellence; CMCH, Chittagong Medical College and Hospital; cDNA, Complementary DNA; ER+, Estrogen receptor-positive; GEPIA, Gene Expression Profiling Interactive Analysis; GLI, Glioma-associated oncogene; Hh, Hedgehog; HER2+, Human epidermal growth factor receptor-positive 2; mRNA, messenger RNA; METTL3, Methyltransferase-like 3; NCBI, National Center for Biotechnology Information (NCBI); m6A, N6-methyladenosine; PCR, Polymerase chain reaction; PTCH, Patched; PR+, Progesterone receptor-positive; qRT-PCR, Quantitative real-time PCR; SMO, Smoothened; SHH, Sonic Hedgehog; SHH-MB, SHH-Medulloblastoma; TCGA, The Cancer Genome Atlas; TNBC, Triple negative breast cancer.
