# Supplementary material for: METTL3-mediated activation of Sonic Hedgehog signaling promotes breast cancer progression
Source: Front Cell Dev Biol. 2025 Oct 1;13:1674339. doi: 10.3389/fcell.2025.1674339 (PMC12521280; doi:10.3389/fcell.2025.1674339)
Supplement: Supplementary file 1 [file DataSheet1.pdf]

Supplementary Material

Supplementary Figures

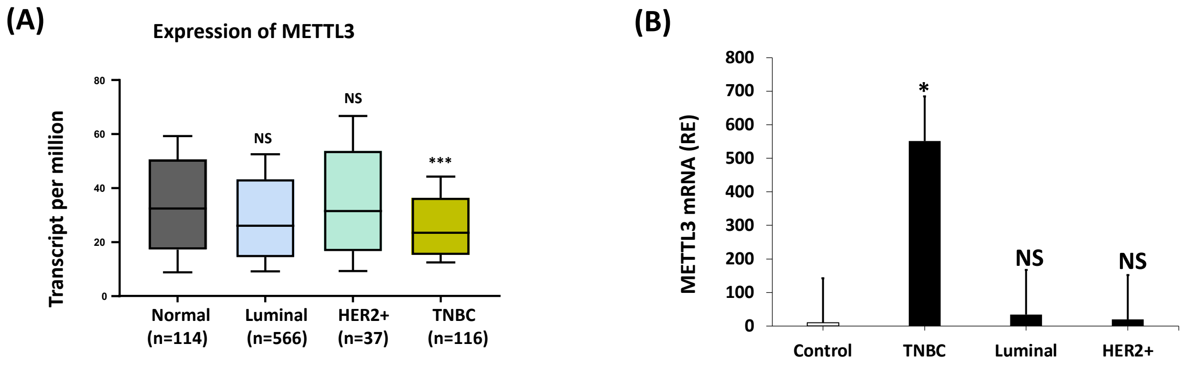

Supplementary Figure S1: Transcript level of METTL3 varies in different subtypes of breast cancer. (A) METTL3 levels are significantly lower in TNBC and luminal breast cancer, while HER2+ show no drifting in TCGA samples. (B) Experimental findings reveal a notable difference in METTL3 expression in TNBC compared to controls, whereas Luminal and HER2+ exhibit no significant change. *p < 0.05 vs. control.

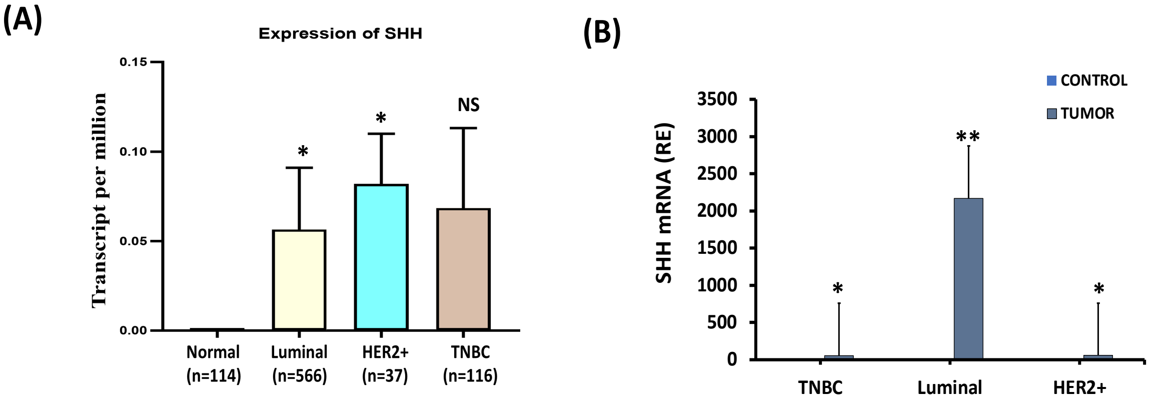

Supplementary Figure S2: TCGA and clinical samples exhibit significant variation in SHH gene expression compared to control samples. (A) The Sonic hedgehog gene demonstrated increased expression across all subtypes of breast cancer; however, this increase was not statistically significant in triple-negative breast cancer TNBC. (B) Analysis of tumor samples revealed that the levels of SHH gene transcripts were significantly higher in all subtypes compared to those found in control samples. **p < 0.01 vs. control.

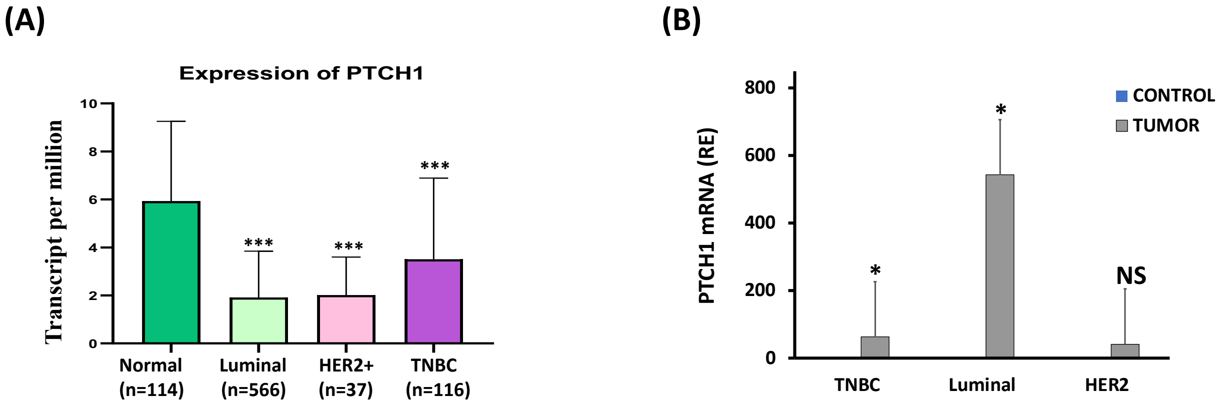

Supplementary Figure S3: Subtype-specific expression pattern of PTCH1 in breast cancer. (A) PTCH1 expression is significantly reduced in all subtypes in the TCGA database. (B) Experimental validation has opposite results for PTCH1 gene and observed a noteworthy expression pattern for TNBC and luminal, in contrast to HER2+. *p < 0.05 vs. control, ***p < 0.001 vs. control.

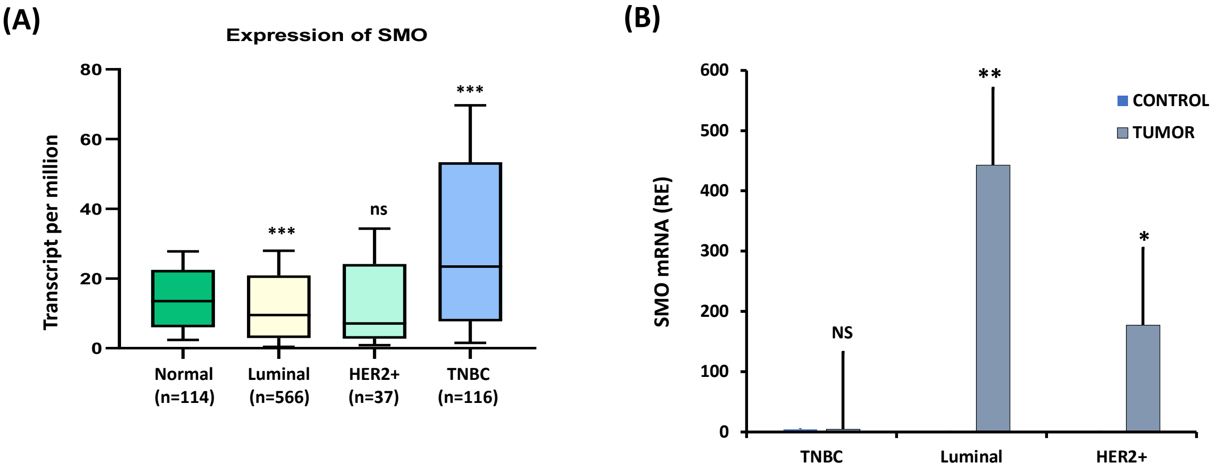

Supplementary Figure S4: Expression patterns of SMO in breast cancer subtypes. (A) TCGA analysis reveals that SMO genes exhibit a significantly lower expression pattern in Luminal breast cancer subtypes; (B) qRT-PCR showing distinct variations in luminal and HER2-positive breast cancer. **p < 0.01 vs. control, ***p < 0.001 vs. control.

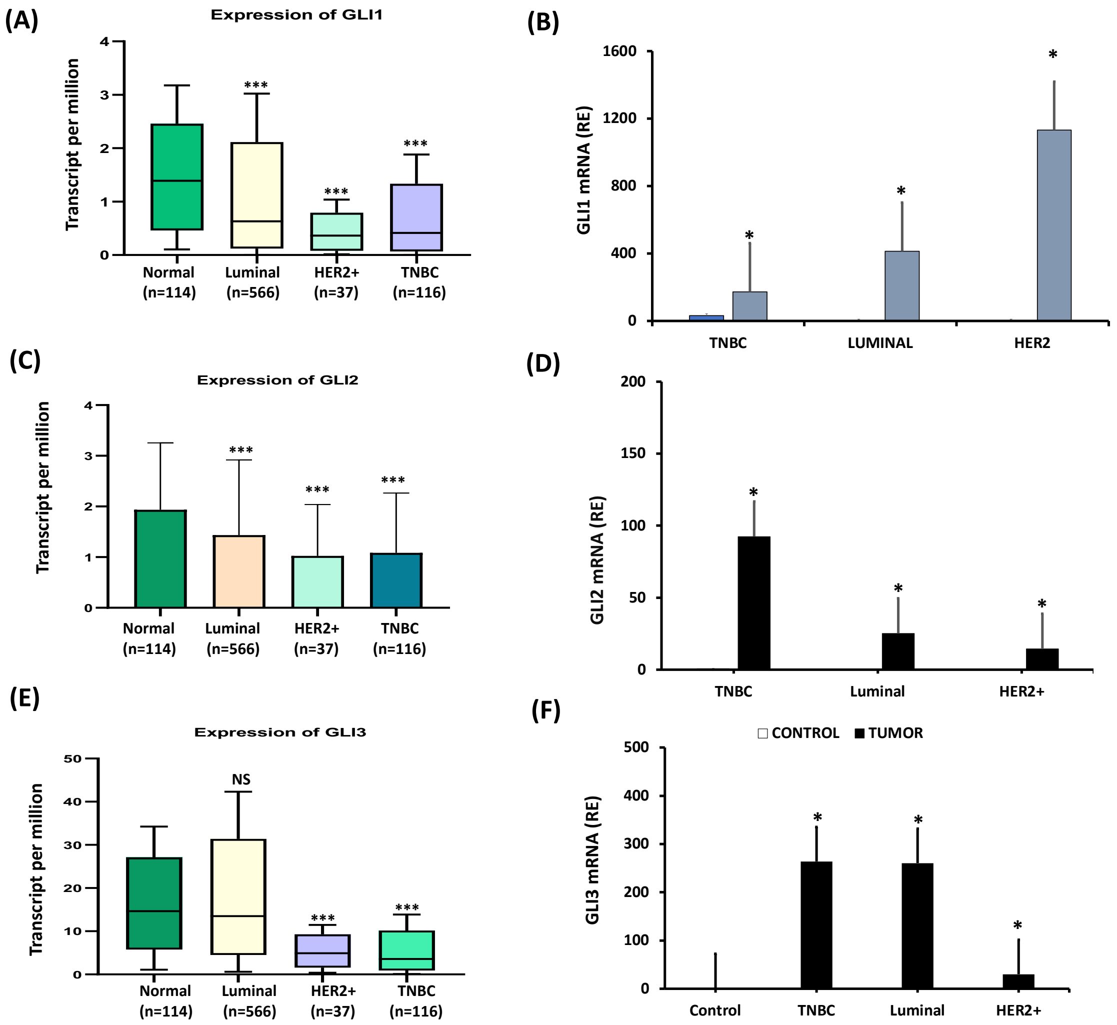

Supplementary Figure S5: Gene expression patterns of GLI transcription factors in breast cancer subtypes. (A) TCGA data demonstrates downregulation of GLI1 across breast cancer subtypes TNBC, Luminal, and HER2+. (B) qPCR analysis reveals increased GLI1 mRNA expression in all types, but not significantly in luminal-type tumors. (C) GLI2 expression is decreased in all breast cancer subtypes, as indicated by data from TCGA databases. (D) Clinical data demonstrate elevated levels of GLI2 compared to control samples. (E) Gli3 mRNA transcript levels are prominently downregulated in TNBC and luminal in the TCGA database. (F) Clinical samples consistently exhibit elevated levels of GLI3 in all subtypes compared to controls. *$p < 0.05$ vs. control, **$p < 0.01$ vs. control ***$p < 0.001$ vs. control.
